# SMC complexes orchestrate the mitotic chromatin interaction landscape

**DOI:** 10.1007/s00294-017-0755-y

**Published:** 2017-09-21

**Authors:** Yasutaka Kakui, Frank Uhlmann

**Affiliations:** Chromosome Segregation Laboratory, The Francis Crick Institute, London, UK

**Keywords:** Chromosome condensation, SMC complex, Chromatin, Cell cycle, Hi-C

## Abstract

Chromatin is a very long DNA–protein complex that controls the expression and inheritance of the genetic information. Chromatin is stored within the nucleus in interphase and further compacted into chromosomes during mitosis. This process, known as chromosome condensation, is essential for faithful segregation of genomic DNA into daughter cells. Condensin and cohesin, members of the structural maintenance of chromosomes (SMC) family, are fundamental for chromosome architecture, both for establishment of chromatin structure in the interphase nucleus and for the formation of condensed chromosomes in mitosis. These ring-shaped SMC complexes are thought to regulate the interactions between DNA strands by topologically entrapping DNA. How this activity shapes chromosomes is not yet understood. Recent high throughput chromosome conformation capture studies revealed how chromatin is reorganized during the cell cycle and have started to explore the role of SMC complexes in mitotic chromatin architecture. Here, we summarize these findings and discuss the conserved nature of chromosome condensation in eukaryotes. We highlight the unexpected finding that condensin-dependent intra-chromosomal interactions in mitosis increase within a distinctive distance range that is characteristic for an organism, while longer and shorter-range interactions are suppressed. This reveals important molecular insight into chromosome architecture.

## Introduction

How chromatin is spatially organized within the cell nucleus and within chromosomes is a fundamental question in cell biology. Centimeter-long DNA molecules change their spatial chromatin organization within micrometer-sized cells during cell cycle progression. In interphase, chromatin is distributed throughout the nucleus to express the genetic information. When cells enter mitosis, chromatin becomes compacted to form mitotic chromosomes. Chromosome condensation, the gross morphological change of spatial chromatin organization in mitosis, is indispensable for the faithful inheritance of genetic information. Structural maintenance of chromosomes (SMC) complexes are large proteinaceous rings that control spatial chromatin organization at various stages during cell growth and differentiation. By topologically entrapping more than one DNA strand within its ring, SMC complexes are thought to mediate interactions between DNA strands for the establishment of chromatin architecture (Uhlmann 2016). Two members of the SMC complex family, cohesin and condensin, play distinct yet overlapping roles in shaping mitotic chromosomes: cohesin holds sister chromatids together (Peters and Nishiyama 2012), while condensin compacts chromatin (Hirano 2016). A third member of SMC family, the Smc5/6 complex is involved in DNA recombination. Its contribution to chromosome architecture is less well understood (Jeppsson et al. 2014). Condensin plays a key role in chromosome condensation, since mitotic chromosome-like structures can be reconstituted by condensin in vitro even in the absence of histones, which form the nucleosome units of chromatin (Shintomi et al. 2017). How condensin promotes mitotic chromosome formation is a topic of great current interest.

Chromosome conformation capture is a powerful technique to investigate spatial chromatin organization (Dekker et al. 2013). Using this technique, spatial information of chromatin interactions is obtained from crosslinked chromatin followed by DNA fragmentation and ligation. High throughout sequencing-based chromosome conformation capture, Hi-C, is able to capture spatial proximities of chromatin in a genome-wide manner (Lieberman-Aiden et al. 2009). Recent Hi-C studies have revealed that chromatin within the interphase nucleus forms domain structures at different size ranges, such as topologically associating domains (TADs) as well as A and B compartments in higher eukaryotes (Dixon et al. 2012; Lieberman-Aiden et al. 2009). These domain structures are controlled by cohesin together with CTCF, the sequence-specific CCCTC-binding factor, to regulate gene expression (Rao et al. 2014; Sofueva et al. 2013). Similarly, cohesin-mediated smaller chromatin domains, called globules, can be seen in fission yeast interphase nucleus (Mizuguchi et al. 2014). In this review, we summarize recent findings on mitotic chromatin architecture in different eukaryotes and discuss how SMC complexes contribute to chromosome condensation.

## Chromatin interactions that convert interphase nuclei into mitotic chromosomes

**Fig. 1:**
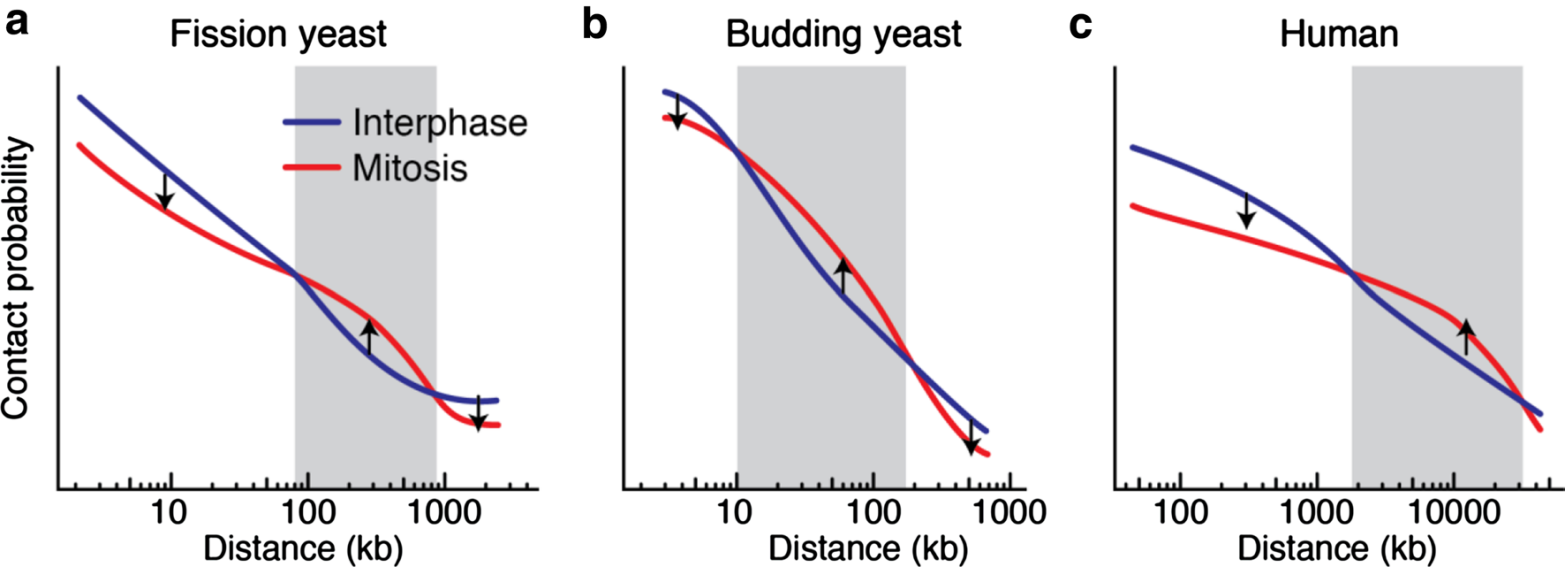
Contact probabilities as a function of genomic distance in three organisms. **a**–**c** Contact probabilities in interphase (blue) and in mitosis (red) are shown as a function of genomic distance in fission yeast (**a**), budding yeast (**b**) and human cells (**c**). The gray squares highlight the area enriched for chromatin interactions in mitosis. Arrows indicate changes of contact probabilities between interphase and mitosis

Recent Hi-C results have illustrated the dramatic alteration of chromatin organization during cell cycle progression in several species (Gibcus et al. 2017; Kakui et al. 2017; Lazar-Stefanita et al. 2017; Nagano et al. 2017; Naumova et al. 2013; Schalbetter et al. 2017). In fission yeast, chromatin interactions are enriched within local areas in the interphase nucleus, whereas chromatin interactions extend towards larger distances in mitotic chromosomes (Kakui et al. 2017). Plotting contact probabilities as a function of genomic distance reveals that contact probabilities decrease as genomic distance between two chromosomal loci increases. This is true both in interphase and mitosis. However, comparing interphase contact probabilities with those in mitosis reveals a relative increase of longer-range interactions at the expense of local chromatin contacts (Fig. 1a). The graph illustrates that chromatin contacts shorter than 90 kb, or greater than 900 kb, are more frequent in interphase as compared to mitosis. In contrast, mitotic chromatin contacts are enriched over distances ranging from 90 to 900 kb (Fig. 1a, gray box). In budding yeast, increased contact probabilities in mitosis are seen in a range up to 100 kb (Schalbetter et al. 2017), while reduced local chromatin contacts are observed in the area below 10 kb (Lazar-Stefanita et al. 2017 and our unpublished observations). Combining these data, Fig. 1b shows qualitatively similar behavior of contact probabilities as a function of genomic distance in budding yeast when compared to fission yeast. However, the distance range enriched for mitotic chromatin contacts are quantitatively different, being shorter in budding yeast (compare the distance ranges highlighted as gray boxes in Fig. 1a and b). In human cells, a reduction of short-range contacts and a corresponding increase of longer-range chromatin interactions in mitosis have also been observed (Fig. 1c) (Naumova et al. 2013). In the case of human cells, the genomic distances that are enriched for contacts in mitotic chromatin are much longer than in either fission yeast or budding yeast (Fig. 1c, gray box). Single-cell Hi-C in mouse embryonic stem cells confirms these cell cycle-dependent changes of chromatin interactions (Nagano et al. 2017). Therefore, the distance range of chromatin contact enrichment in mitosis is characteristic for each organism and appears to be related to the overall chromosome size (Fig. 1, Compare gray boxes). As chicken DT40 cells enter mitosis, a band of new mitotic contacts shifts towards longer distances over time (Gibcus et al. 2017), accompanied by shortening and thickening of chromosome arms. The relationship between mitotically enriched chromatin interactions and chromosome size features will be interesting to explore. Does the interaction size range define chromosome shape, or does chromosome shape constrain the interactions? Answers to these questions will further our understanding of chromosome condensation.

A notable feature of mitotic chromosomes is a steep drop of contact probabilities at very large genomic distances, over 10 Mb in human cells (Naumova et al. 2013). Mitotic reduction of the longest-range interactions can also be seen in both fission yeast and budding yeast (Kakui et al. 2017; Schalbetter et al. 2017). It is probably a reflection of mitotic chromosome arm stiffening, which is expected to disfavor longest-range interactions. An important point to keep in mind, when comparing interaction frequencies in interphase and mitosis, is that Hi-C reports on relative interaction frequencies, not on absolute contact frequencies. To calibrate interaction changes, we have quantitatively analyzed chromatin contact frequency changes by 3C followed by quantitative real-time PCR. Cytological observations served as an additional means to validate interaction frequency changes. This confirmed an absolute increase of mitotic interactions in the range between 90 and 900 kb in fission yeast, while local interactions are indeed reduced (Kakui et al. 2017). This portrays mitotic chromosome formation as the consequence of increased interactions in a specific size range, accompanied by a quantitative reduction of short- and longest-range interactions.

The mitotic reduction of local chromatin contacts comes as a surprise, as one would expect that all DNA sequences come closer together in a condensed chromosome. However, new interactions at longer distances will restrict the freedom of movement of the chromatin chain, thereby reducing the probability of local interactions. Consistently, local chromatin motility becomes constrained in mitosis (Kakui et al. 2017). It will be important to investigate whether a quantitative reduction of short-range chromatin interactions occurs in organisms other than fission yeast. If confinement of local chromatin motility is a general feature of mitotic chromosomes, its consequences for maintenance and reprogramming of the gene expression program during mitosis will be important to examine.

## The contribution of SMC complexes to chromosome condensation

**Fig. 2:**
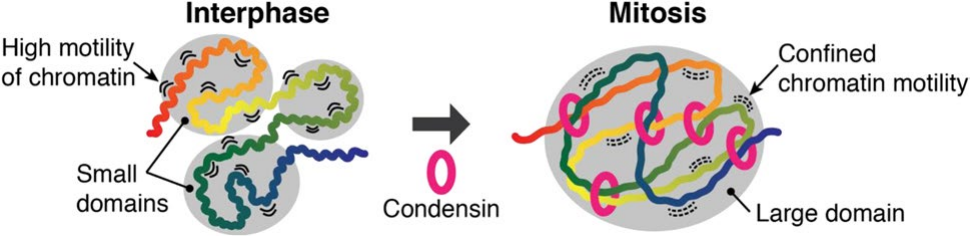
Schematic of condensin-mediated chromosome condensation. Many small domains are formed in interphase. Condensin replaces local contacts with longer-range interactions, resulting in the formation of larger domains. In parallel, condensin confines chromatin motility. Domains are shown as gray circles. One-dimensional position of chromatin is shown as color gradient

All the above described changes of chromatin contacts in fission yeast mitosis are dependent on condensin (Fig. 2) (Kakui et al. 2017). Furthermore, condensin-enriched sites preferentially interact with each other, although widespread contacts also extend to parts of chromosomes with less prominent condensin binding. These results are consistent with condensin recruitment to highly specific transcribed genes (D’Ambrosio et al. 2008; Nakazawa et al. 2015; Robellet et al. 2017; Schmidt et al. 2009; Sutani et al. 2015), and the condensin-dependent interactions between PolIII transcribed genes in interphase (Hausler et al. 2008; Iwasaki and Noma 2016). Fission yeast condensin not only locates at chromatin domain boundaries, but also promotes fusion of interphase domains to generate larger domains in mitosis (Fig. 2). Condensin-dependent replacement of interphase chromatin contacts to shape mitotic chromosomes is also seen in chicken DT40 cell (Gibcus et al. 2017), suggesting that condensin converts interphase chromatin organization into mitotic chromosomes also in higher eukaryotes. In addition to condensin, cohesin provides a good portion of intra-chromosomal chromatin contacts in budding yeast mitosis (Lazar-Stefanita et al. 2017; Schalbetter et al. 2017). This is consistent with the role of budding yeast cohesin in chromosome condensation (Guacci et al. 1997). Budding yeast condensin is most highly concentrated along the rDNA repeats on chromosome XII, that it helps to compact in mitosis (Schalbetter et al. 2017; Sullivan et al. 2004). In addition, condensin contributes together with cohesin to chromosome arm compaction (D’Ambrosio et al. 2008; Strunnikov et al. 1995). Circular chromosome conformation capture (4C) revealed that condensin binding sites on the chromosome V long arm engage in increased contacts with chromatin loci located within approximately 100 kb from the viewpoints in mitosis, an increase that depends on the condensin subunit Brn1 (Cheng et al. 2015). The distance of increased chromatin contacts detected by 4C is consistent with the range of mitotic contact probability increase detected by Hi-C, revealing how budding yeast condensin likely contributes to compact chromosome arms. The budding yeast cohesin subunit Scc1 is cleaved in anaphase, such that there is barely any functional cohesin in G1 phase. This could explain cohesin’s bigger impact on chromosome condensation in budding yeast mitosis, compared to other organisms in which cohesin undergoes less dramatic alterations during the cell cycle. Presumably, both cohesin and condensin contribute to chromosome architecture in overlapping ways by setting up local chromatin domains and by controlling their fusion into larger assemblies during chromosome condensation. Their relative contribution may differ between organisms, dependent on the size of chromosomes. As fission yeast chromatin forms 50–100 kb sized globules in interphase, that depend on cohesin (Mizuguchi et al. 2014), it will be of interest to examine how cohesin contributes to interactions in this size range in mitosis in this organism. Whether and how the Smc5/6 complex contributes to the intra-chromosomal interaction spectrum in interphase and mitosis remains to be seen.

The recent Hi-C experiments unveiled that SMC complexes drive a dramatic reorganization of chromatin contacts as cells enter mitosis. They did not elucidate the mechanism of how SMC complexes mediate chromatin interactions. Two proposed models how SMC rings modulate chromatin contacts are the stabilization of stochastic pairwise interactions and loop extrusion, two models that need not be mutually exclusive (Cheng et al. 2015; Fudenberg et al. 2016). Higher resolution Hi-C datasets, coupled with computational modeling, should shed light on the mechanism of how SMC complexes control chromosome formation. A molecular understanding of how chromosomes take shape might soon be in sight.
